# Decoding elastin–collagen resemblance in keloid scar through label-free imaging and machine learning

**DOI:** 10.1117/1.JBO.31.3.036005

**Published:** 2026-03-14

**Authors:** Chuncheng Wang, Jia Meng, Lingxi Zhou, Lingmei Chen, Shuhao Qian, Rushan Jiang, Changyong Chen, Lu Yang, Lu Chen, Wei Zhou, Zhihua Ding, Shuangmu Zhuo, Zhiyi Liu

**Affiliations:** a Zhejiang University, State Key Laboratory of Extreme Photonics and Instrumentation, College of Optical Science and Engineering, International Research Center for Advanced Photonics, Hangzhou, China; b Zhejiang University, Department of Gynecology and Obstetrics, The First Affiliated Hospital, College of Medicine, Hangzhou, China; c Jimei University, School of Science, Xiamen, China; d Zhejiang University, Jiaxing Research Institute, Intelligent Optics and Photonics Research Center, Jiaxing, China

**Keywords:** bio-photonics, extracellular matrix, elastin fiber, collagen fiber, keloid scar

## Abstract

**Significance:**

Label-free imaging of keloid scar tissues and inter-channel characterization of fibrous structures provide insights for understanding the process of extracellular matrix (ECM) remodeling during human skin aberrant wound healing.

**Aim:**

Multiphoton microscopy imaging is used for *ex vivo* human skin samples, based on endogenous signals of elastin and collagen fibers, and an algorithm is designed to quantify the resemblance in morphology and structure between the two fiber components.

**Approach:**

Based on two-photon excitation fluorescence images of elastin fibers and second harmonic generation images of collagen fibers in normal, keloid, and adjacent skin samples, a parameter termed “resemblance metric” (RM) is developed to quantify the morphological and organizational similarity of the two fiber components within the human keloid scar model. The application potential of this method is demonstrated by identifying inter-heterotypic-fibrous resemblance features of three tissue types with high sensitivity.

**Results:**

Keloid scar tissues exhibit the highest elastin–collagen resemblance level, and adjacent tissues are the most heterogeneous. Using this parameter, adjacent tissues are identified with an accuracy higher than 98%.

**Conclusions:**

The high sensitivity of RM in interpreting the elastin–collagen resemblance within the human keloid scar model reveals a perspective in understanding the mechanism of ECM remodeling.

## 1 Introduction

Human keloid scars represent a pathological skin disorder characterized by aberrant wound healing responses. Unlike normal scars, keloids demonstrate progressive expansion beyond initial wound margins through invasive peripheral growth without regression or contraction.^1^^,^^2^ Besides, these abnormal appearances are often associated with pain, pruritus, cosmetic concerns, and sometimes paraneoplastic risk.^3^^–^^6^ Keloids with suspicious clinical features require biopsy and histopathological examination for diagnosis confirmation.^7^^,^^8^ This process primarily relies on staining techniques^9^^–^^11^ to enhance tissue contrast for microscopic analysis. However, these procedures involve time-consuming tissue processing^12^ and might introduce inter-observer disagreement due to subjectivity that leads to potential diagnostic variability. These limitations highlight the need for a rapid and label-free approach to improve keloid assessment with higher consistency and greater efficiency.

Abnormal remodeling and reorganization of the extracellular matrix (ECM) are essential contributors to abnormal scar formation.^13^ As the ECM is an important target of keloid-induced changes, proper assessment of ECM microstructures might provide promising biomarkers to identify keloid scars. Among all ECM components, elastin and collagen fibers are two key fibrous structures that offer resilience and tenacity, respectively.^14^^,^^15^ Multiphoton microscopy (MPM), which leverages two-photon excitation fluorescence (TPEF) and second harmonic generation (SHG), offers distinctive advantages for dermatological research. It enables label-free, high-resolution imaging of unstained tissues in their native state.^16^^,^^17^ The intrinsic contrast mechanisms of MPM are ideal for skin imaging because elastin fibers (as natural fluorophores) exhibit endogenous characteristic TPEF signals, whereas dermal collagen fibers produce strong SHG signals (due to its noncentrosymmetric structure), allowing their simultaneous visualization. These attributes have established MPM as an irreplaceable tool in dermatology, facilitating investigations into skin physiology, such as aging and photoaging processes,^18^^–^^20^ and pathology, such as skin neoplasia.^21^^,^^22^ Besides, this nondestructive imaging modality is also applicable for instant imaging after fresh excision from skin, indicating its growing potential for clinical translation. Therefore, MPM imaging serves as a reliable tool to characterize distinctive morphological alterations of elastin and collagen networks at the microscale, offering insights for further diagnostic and therapeutic advances.

Another distinct feature of a keloid scar is the increased tissue stiffness at the macroscale. Generally, keloid tissues are ∼10× stiffer than normal skin tissues.^23^ On the microscale, this variation can be attributed to the remodeling of the ECM, especially in collagen disorders.^24^ For example, a typical feature of keloid scar is the dysregulated keloid-derived fibroblasts producing excessive fibrillar ECM deposition during recovery from cutaneous injury, particularly collagen.^25^ Present studies mainly focused on interpreting keloid stiffening through genetic expression and molecular bioscience, with little attention paid to the direct evidence of alteration of ECM components. Meanwhile, the structural remodeling of elastin and collagen fibers within the ECM of keloid scar remains unclear. Therefore, there is an urgent need for an automated method to comprehensively characterize the microstructure of keloid scars through interpretable analysis of both elastin and collagen fiber features.

In this study, we developed a label-free MPM imaging platform with automated analysis of elastin and collagen fibers to examine the structural resemblance between the two ECM fibrous components within the human keloid scar model. First, the MPM module was used to image the two key ECM structures—elastin and collagen fibers—in *ex vivo* samples. Next, we extracted a series of morphological features to generate pixel-wise feature maps. Building on this, a novel parameter termed the “resemblance metric” (RM) was introduced to quantify the morphological similarity between these two key ECM components. Subsequently, the mean and standard deviation (SD) of all extracted features were evaluated using the XGBoost algorithm to establish the optimal feature subset. Finally, the optimal feature subset—comprising RM mean and RM SD—was highlighted for its high sensitivity in distinguishing among normal, keloid, and adjacent tissues, thereby suggesting elastin–collagen resemblance as a novel possibility for understanding ECM remodeling.

## 2 Materials and Methods

### 2.1 Study Design

**Fig. 1:**
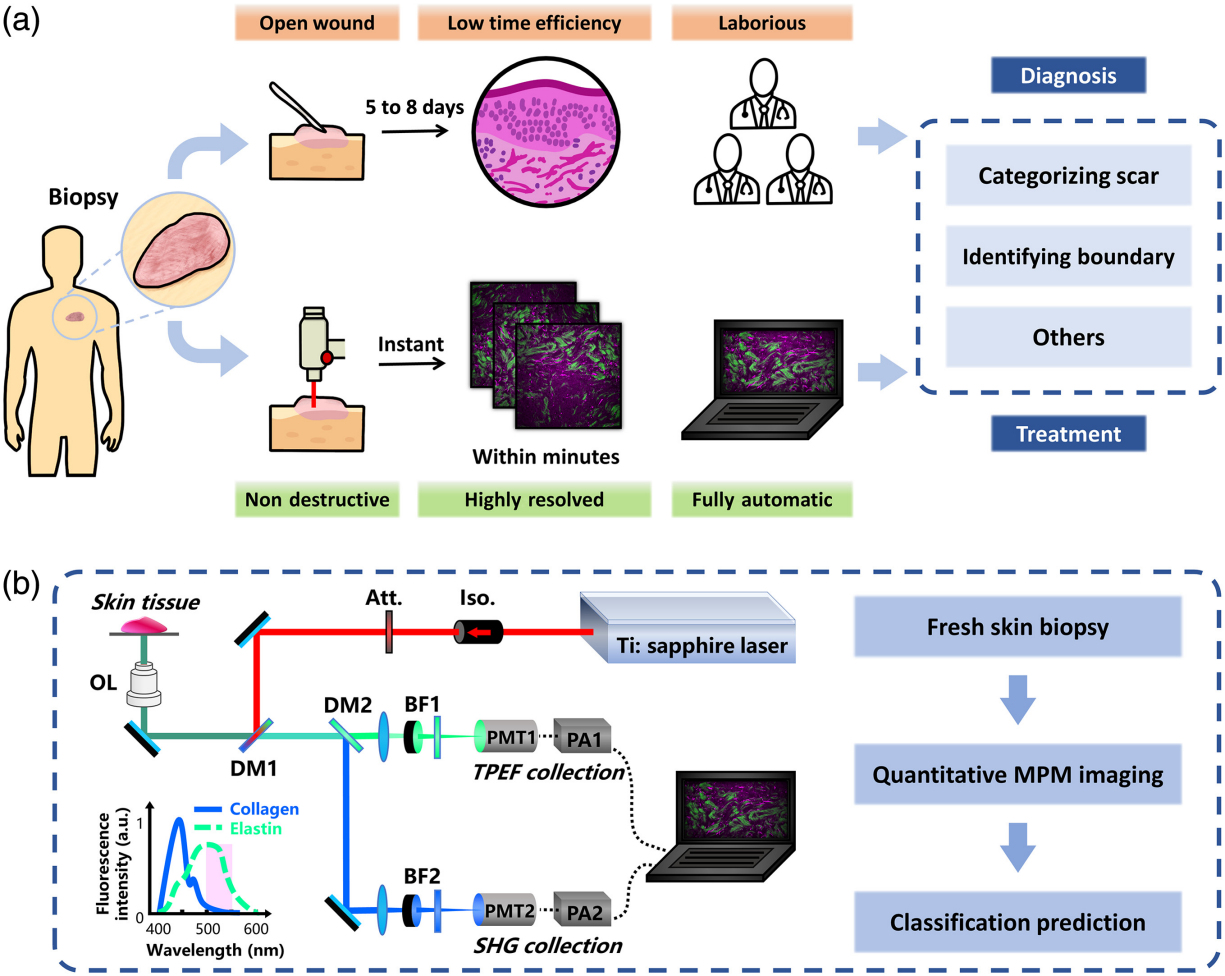
Comparison between the traditional pathology approach and the automated approach. (a) Comparison between clinically applied traditional pathology approach (up) and our proposed automated approach (down), where excised tissues were instantly imaged instead of cumbersome traditional histopathological procedures. Images were then analyzed with machine-learning-based feature analysis. (b) Configuration of quantitative multiphoton microscopy. Inset: Fluorescence spectra of elastin and collagen samples by excitation at 800 nm. The light magenta rectangle corresponded to the collected emission band with a large amount of fluorescence signals from elastin fibers and minimum fluorescence signals from collagen fibers. Iso., isolator; Att., attenuator; DM, dichroic mirror; OL, objective lens; BF, bandpass filter; PMT, photomultiplier tube; PA, preamplifier.

The diagnosis of keloid scars currently relies on conventional histopathological techniques, which remain time-consuming, laborious and specialized expertise required. These limitations call for the development of more precise and efficient diagnostic solutions [Fig. 1(a)]. To this end, we developed an automated platform capable of accurately detecting the features among normal, keloid, and adjacent skin tissues. This system integrated quantitative nonlabeling multiphoton imaging with machine-learning algorithms to select an optimal feature subset to provide fast, objective, reproducible tissue characterization readouts.

### 2.2 *Ex Vivo* Human Keloid Sample Preparation

A total of 34 human keloids, 27 adjacent samples, and 47 normal skin samples were obtained from 56 patients undergoing reconstructive surgery, with informed consent from all patients themselves and/or their legal guardians. The ethnicity of patients involved in this study were all Han Chinese, with demographic distribution (age and gender) and anatomical location distribution listed in Table S1 in the Supplementary Material. Immediately after excision, tissues were snap-frozen in liquid nitrogen (−196°C) to preserve native morphology and prevent protein denaturation. For MPM imaging, the acquired samples were cut into ∼60  μm thickness, sandwiched between a microscope slide and cover glass, and hydrated with phosphate-buffered saline (PBS) to minimize dehydration artifacts during imaging. After imaging, the sections were conducted histological staining, and their categories were identified by pathologists. The above procedures were approved by the Institutional Review Board of Zhejiang University.

### 2.3 Multiphoton Microscopy Setup

The MPM imaging system [Fig. 1(b)] consisted of an inverted laser-scanning microscope (Zeiss, LSM 510, Oberkochen, Germany) coupled with a tunable mode-locked Ti:sapphire femtosecond laser (700 to 980 nm, 110 fs, 76 MHz pulse rate). A 63× oil-immersion objective (NA 1.4, Plan-Apochromat) was used for high-resolution imaging. Using 800 nm as the excitation wavelength, elastin and collagen fibers were imaged via backward-detected TPEF and SHG signals, respectively. TPEF signals (430 to 697 nm, elastin) and SHG signals (398 to 409 nm, collagen) were isolated using two distinct spectral channels, with pseudo color-coding (magenta for elastin and green for collagen) to enhance contrast. Images were acquired with a pixel dwell time of 2.56  μs and at a field of view of 210×210  μm. The imaging process was monitored in real time on the screen, allowing immediate reacquisition at a new location if image distortion occurred, ensuring optimal resolution and contrast.

### 2.4 Feature Extraction

There were two categories of features to be extracted from the images of elastin and collagen fibers for analysis, quantified by high-precision, pixel-wise algorithms specifically designed for fibrous structures. One category was the morpho-structural features of fibers themselves, including local coverage (LC),^26^^,^^27^ orientation (Ori, designated by the azimuthal angle, Fig. S1 in the Supplementary Material),^28^ directional variance (DV),^29^ and waviness (Wav).^30^ In addition, the thickness^31^ of the elastin fiber was estimated. After extraction, these features were visualized in pseudo color-coded maps on a pixel-wise basis. The basic algorithmic principles of these features are detailed in the Supplementary Material. The other category of parameter was the newly established RM based on the above-mentioned morphological features. To be noted, RM comprehensively and quantitatively assessed the morphological and organizational similarity between elastin and collagen fibers from multiple perspectives, including distance, local coverage, orientation, directional variance, and waviness, resulting in a normalized pixel-wise map.

### 2.5 Resemblance Metric (RM)

**Fig. 2:**
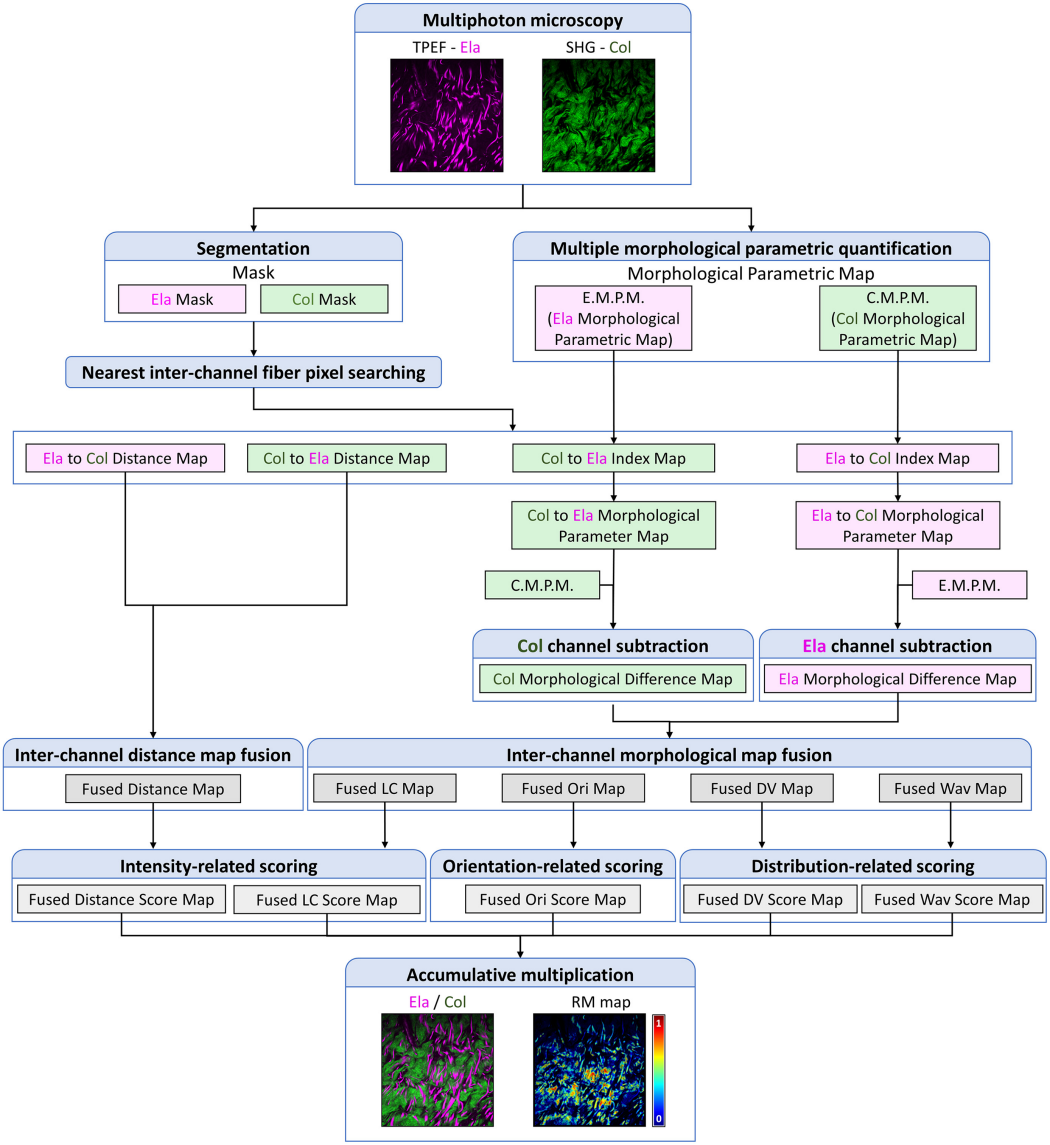
Flowchart upon establishment of elastin–collagen RM based on multiple morphological feature maps of multiphoton images. Ela, elastin; Col, collagen; LC, local coverage; Ori, orientation; DV, directional variance; Wav, waviness; RM, resemblance metric.

The RM quantified elastin–collagen morpho-structural similarity by integrating the differences of nearest inter-fiber distances with comprehensive morphological features (local coverage, orientation, directional variance, and waviness) of relative pixel pairs. Higher RM values indicated a more similar level of spatial morphology distribution between the measured fibers. The flowchart for RM establishment is shown in Fig. 2.

**Fig. 3:**
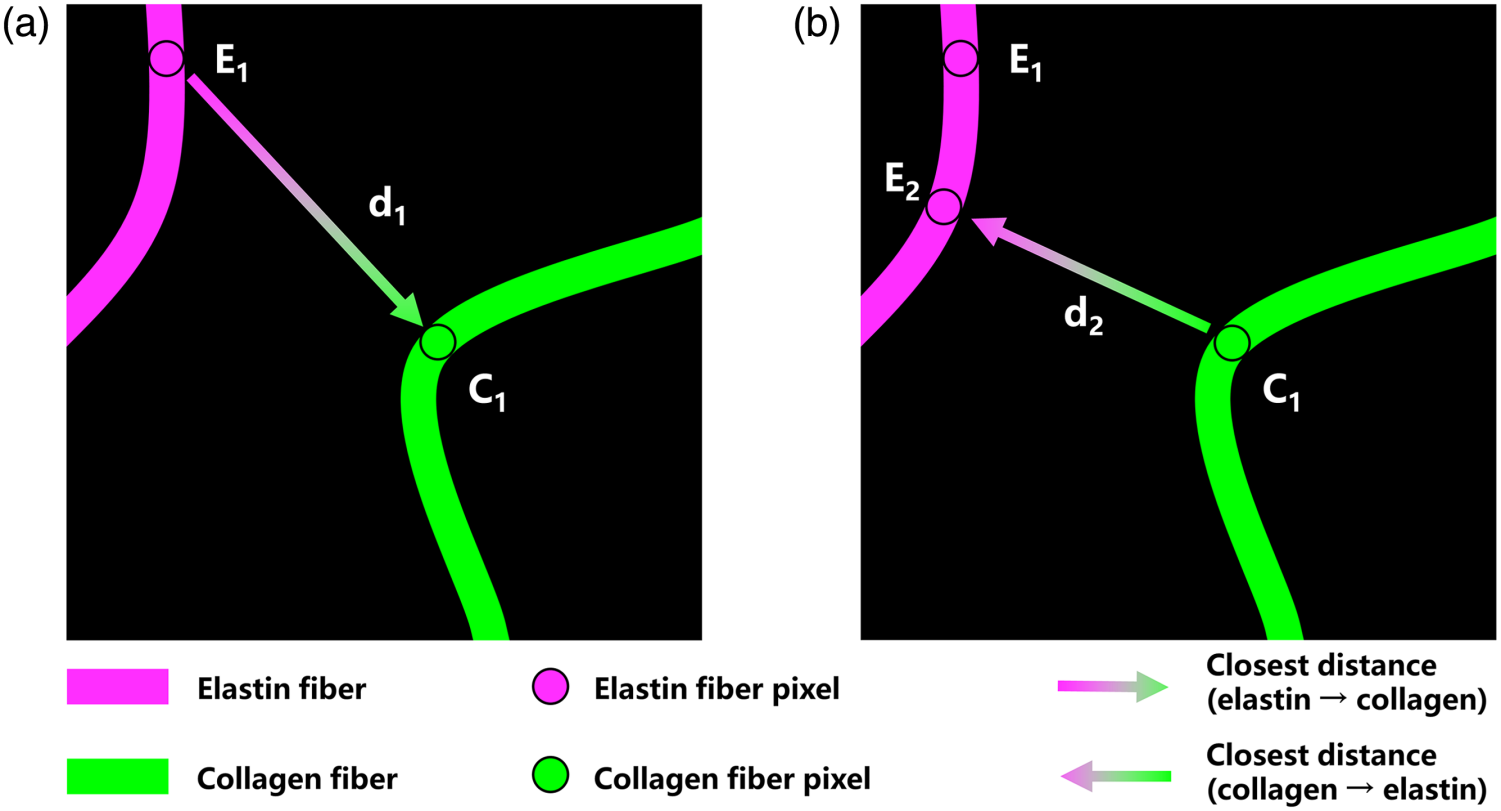
Illustration of bidirectional analysis for asymmetric inter-channel nearest-neighbor relationship. (a) The closest distance from elastin pixel $E_{1}$ to its nearest collagen pixel $C_{1}$. (b) The closest distance from collagen pixel $C_{1}$ to its nearest elastin pixel $E_{2}$, demonstrating that $d_{1}\neq d_{2}$ and $E_{1}\neq E_{2}$.

The RM calculation involved a comprehensive two-dimensional (2D) quantification of elastin and collagen fiber features (including local coverage $L_{2D}$, azimuthal angle θ, directional variance $V_{2D}$, and waviness $W_{2D}$). For each elastin pixel, we found its nearest collagen pixel (and vice versa), generating a minimum distance matrix ($d_{2D}$) and an inter-channel index matrix for both channels, respectively. Here, bidirectional analysis was necessary because the nearest-neighbor relationship is normally asymmetric. As illustrated in Fig. 3, if a collagen pixel $C_{1}$ was the closest one to an elastin pixel $E_{1}$, the elastin pixel $E_{1}$ might not be the reverse closest elastin pixel from the collagen pixel $C_{1}$ and vice versa. This asymmetric fact highlighted the importance of bidirectional computation for capturing inter-channel fibers’ resemblance in a real environment.

To begin with, feature differences between closest paired inter-channel pixels were then taken as the absolute value based on the subtraction between their respective feature maps:

$$M_{\mathrm{SElaF}}=|M_{\mathrm{ElaF}}-M_{\mathrm{ElaF}-\mathrm{CloColF}}|,$$ (1)

$$M_{\mathrm{SColF}}=|M_{\mathrm{ColF}}-M_{\mathrm{ColF}-\mathrm{CloElaF}}|,$$ (2)

where $M_{\mathrm{ElaF}}$ and $M_{\mathrm{ColF}}$ are raw morphological feature maps of elastin and collagen channel, respectively, $M_{\mathrm{ElaF}\text{-}\mathrm{CloColF}}$ and $M_{\mathrm{ColF}\text{-}\mathrm{CloElaF}}$ are the feature maps of the closest pixel from the other channel. In particular, the subtraction matrix of the orientation feature was converted to its complementary angle when exceeding 90 deg, and the subtraction matrices also included a distance feature map, with the raw “morphological feature map” being all zero. For each feature, the two subtraction matrices $M_{\mathrm{SElaF}}$ and $M_{\mathrm{SColF}}$ were then fused into fused feature maps through the following equations:

$$M_{\mathrm{FF}}=(m_{E}-m_{E\wedge C})M_{\mathrm{SElaF}}+(m_{C}-m_{E\wedge C})M_{\mathrm{SColF}}+m_{E\wedge C}(\frac{M_{\mathrm{SElaF}}+M_{\mathrm{SColF}}}{2}),$$ (3)

$$m_{E\wedge C}=m_{E}\wedge m_{C},$$ (4)

where $m_{E}$ and $m_{C}$ were binary masks of elastin and collagen fibers. After inter-channel fusion, each fused feature map utilized a specialized scoring strategy to be converted into normalized feature resemblance maps. At first, the scoring expression for the fused distance difference matrix $M_{\mathrm{FD}}$ was

$$S_{\mathrm{FD}}=\sqrt{\frac{1}{1+\frac{M_{\mathrm{FD}}}{D_{\text{uplim}}}(e-1)}},$$ (5)

where $D_{\text{uplim}}$ is referred to as the maximum value of the elastin thickness feature map, as RM would decay rapidly when the inter-fiber distance increases, and e is the natural constant. Collagen fiber thickness was not referred to due to its characteristic entanglement, preventing individual fiber resolution. Similarly, the scoring expression for the fused feature matrix of local coverage difference $M_{\mathrm{FL}}$ (as the other pixel-intensity-based feature) was

$$S_{L}=\sqrt{\frac{1}{1+M_{\mathrm{FL}}(e-1)}}.$$ (6)

For the fused orientation difference matrix category, the scoring expression was

$$S_{O}=\cos(M_{\mathrm{FO}}).$$ (7)

For fused difference matrices concerning features reflecting collective distributions ($M_{\mathrm{FCD}}$), including dictional variance maps ($M_{\mathrm{FV}}$) and waviness maps ($M_{\mathrm{FW}}$), the scoring expression was

$$S_{\mathrm{CD}}=\frac{1}{2}\frac{e^{-C(M_{\mathrm{FD}}-1/2)}-e^{C(M_{\mathrm{FD}}-1/2)}}{e^{-C(M_{\mathrm{FD}}-1/2)}+e^{C(M_{\mathrm{FD}}-1/2)}},$$ (8)

where C is an empirical coefficient valued 4 here, to enhance contrast for intermediate values. This coefficient also allowed adjustment when translated to other disease models. Finally, RM was integrated by combining all normalized score matrices through Hadamard product accumulation.

$$RM=S_{D}S_{L}S_{O}S_{V}S_{W}.$$ (9)

RM ranged from 0 to 1, with higher values indicating greater elastin–collagen morphological and organizational resemblance. Here, the weight was assigned equally (as 1) to all features in this study, which was adjustable when translated to other disease models.

### 2.6 Spectra Measurement for Elastin and Collagen Fibers

The spectral measurements of elastin and collagen fibers followed an established protocol.^32^ Elastin samples consisted of human lung-derived powder (E7152, Sigma-Aldrich, St. Louis, Missouri, United States) obtained through non-degradative extraction. The elastin powder was rehydrated with water and mounted between a microscope slide and a coverslip. Collagen samples were prepared as fibrillar gels using type I collagen isolated from rat tail tendons. Measurements were performed using the custom system described above at an 800 nm excitation wavelength, matching the tissue imaging parameters. TPEF and SHG signals were acquired and normalized to maximum intensity for spectral comparison [inset in Fig. 1(b)].

### 2.7 Optimal Feature Subset Selection

To optimize the classification performance, we implemented Extreme Gradient Boosting (XGBoost), a gradient boosting framework, to optimize the optimal feature subset selection. A combined usage of Bayesian optimization and XGBoost’s built-in early stopping mechanism was utilized to automate XGBoost hyperparameter tuning, restraining overfitting on our dataset. Given the limited data size, we applied k-fold cross-validation (k=5) with balanced class distribution in each fold.

### 2.8 Statistical Analysis

The statistical analysis was based on the extraction of the mean (indicating the comprehensive level) and standard deviation (SD, indicating scattering level) of feature maps. One-way ANOVA with Tukey’s post-hoc test was performed using JMP. K-nearest neighbor (KNN) machine learning classifier evaluated RM’s classification performance for normal/adjacent/keloid classification with one-vs.-rest technique for ROC analysis and leave-one-out (LOO) technique for cross-validated classification accuracy (CVCA).

## 3 Results

### 3.1 Quantitative MPM Imaging Results

**Fig. 4:**
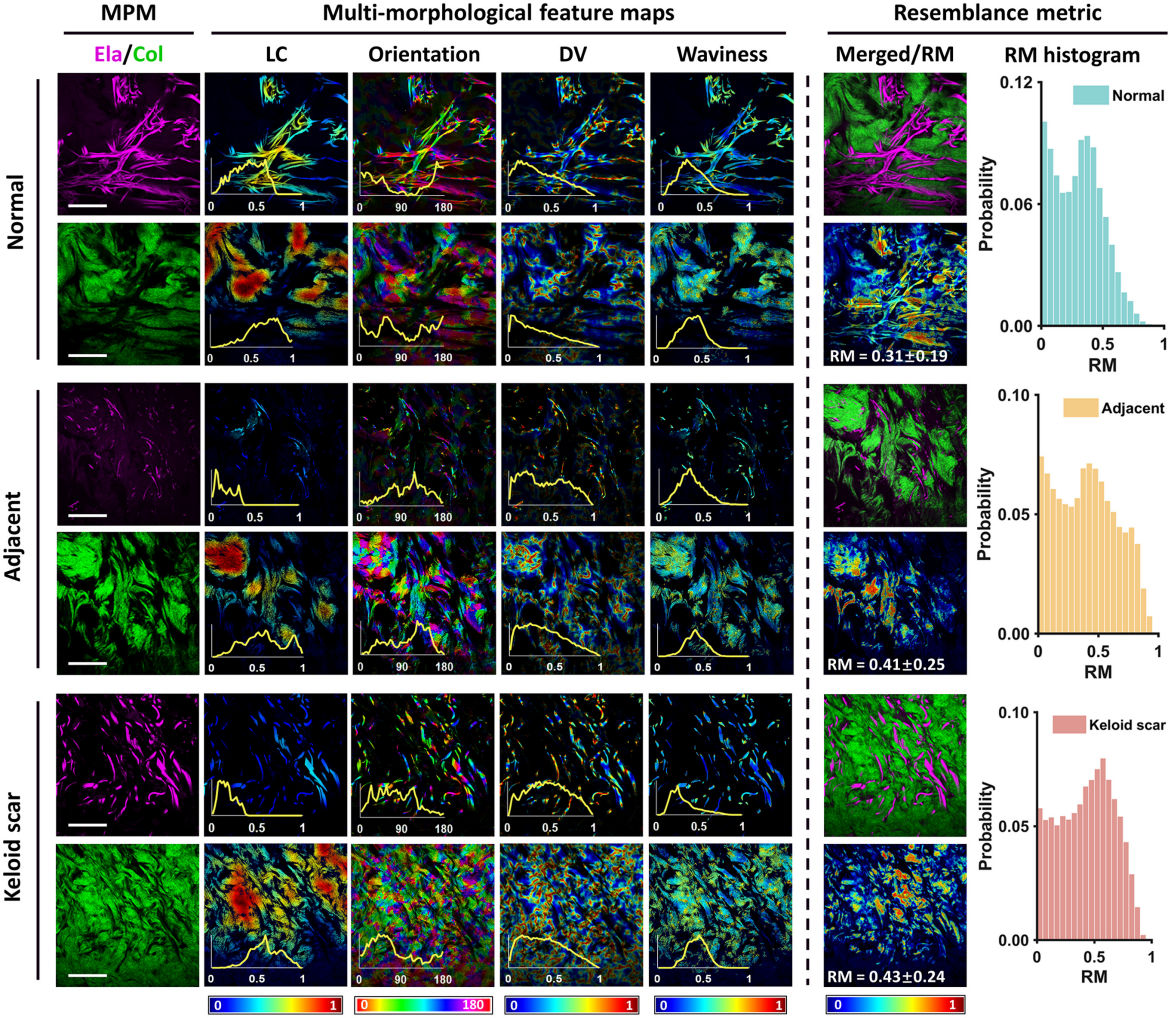
Quantitative multiphoton images on elastin and collagen fibers of human normal, adjacent, and keloid scar tissues concerning pixel-wise maps of local coverage, orientation, directional variance, waviness, and RM, with histogram distributions depicted. MPM, multiphoton microscopy; Ela, elastin; Col, collagen; LC, local coverage; DV, directional variance; RM, resemblance metric. Scale bar, 50  μm.

Representative examples of quantitative MPM imaging results of normal, adjacent, and scar tissues with normalized feature maps of local coverage (LC, with 0 to 1 indicating increasing density), orientation (Ori, within ∼0  deg to 180 deg), directional variance (DV, with 0 to 1 indicating increasing disorder), waviness (Wav, with 0 to 1 indicating increasing curvature), and resemblance metric (RM, with 0 to 1 indicating increasing resemblance) are presented in Fig. 4, with feature histograms also depicted. Thickness maps of elastin channels for each category are shown in Fig. S2 in the Supplementary Material.

As can be seen from Fig. 4, both elastin and collagen fibers in normal tissues exhibited higher density levels compared with adjacent and keloid scar tissues, which could be more intuitively observed from the local coverage maps and related histograms. For the spatial orientation, multipeak distributions were observed in the orientation distribution for both elastin and collagen fibers in three categories, with a higher discrepancy identified in keloid scar tissues. Notably, keloid elastin and normal collagen exhibited the most scattered distributions in three categories, respectively. For the alignment feature, elastin in keloid scar tissues showed the most disordered distribution, and collagen in normal tissues showed the most aligned distribution. For the waviness feature, elastin in keloid scar tissues was observed to be straighter than in the other categories, whereas collagen waviness distributions were comparable in different tissue types. For estimated elastin thickness, elastin in normal and keloid scar tissues showed multipeak distribution, whereas elastin in adjacent tissue was much thinner and more densely distributed (Fig. S2 in the Supplementary Material).

For elastin–collagen RM analysis, the RM feature provided illustrations on both precise spatial mappings (shown in RM maps) and comprehensive distributions (shown in RM histograms). In Fig. 4, morphological feature maps of normal skin tissues showed rather consistent distribution between collagen and elastin fibers, in accordance with the RM map exhibiting a large area of blue hues and the peaks at low RM level in the RM histogram. These results could be attributed to large collagen-intensive areas in the collagen channel. These results also indicated that areas with low elastin–collagen RM level could hardly be reflected by the comparison of morphological feature maps or relative histograms but could be illustrated by the RM histogram peaks and the corresponding RM map. For adjacent tissues, elastin signals were much weaker compared with those in the normal tissue, and a lower elastin–collagen RM level could be confirmed from the maps and histograms of local coverage and directional variance features. However, compared with normal tissues, the closer proximity between the small amount of elastin and the surrounding collagen resulted in the second peak with higher RM values in the RM histogram. For keloid scar tissues, elastin content levels exceeded adjacent tissue but remained below normal levels, whereas the collagen coverage was rather high, especially in the very close proximity of elastin. As a result, although the histograms of morphological features showed a rather distinctive distribution, the two fibers in very proximal distance and perplexing with each other could be recognized in the RM map, resulting in a right-shifted RM histogram distribution following the order of normal, adjacent, and keloid categories.

### 3.2 Reproducibility Assessment of RM Quantification

**Fig. 5:**
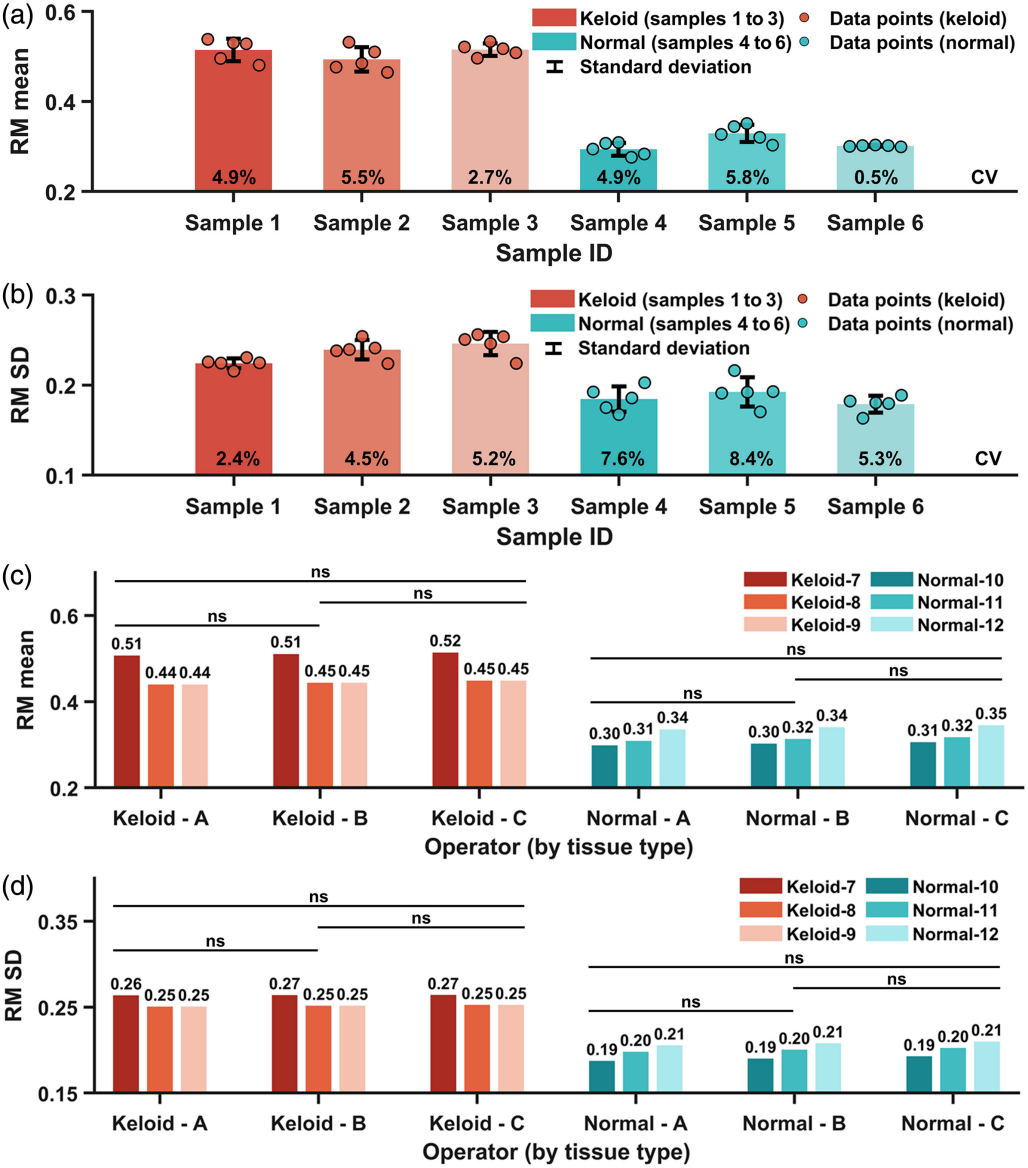
Assessment of intra-sample reproducibility and inter-operator variability in RM quantification. (a) RM mean and (b) RM SD quantification on three keloid and three normal samples for intra-sample reproducibility assessment, with each sample quantified with randomly selected 5 non-overlapping regions. CV, coefficient of variation; SD, standard deviation. (c) RM mean and (d) RM SD quantification on three keloid and three normal samples for inter-operator variability assessment, with each sample quantified by operators A, B, and C. ns, nonsignificant (p>0.05).

The RM method’s intra-sample reproducibility and inter-operator variability were also examined (Fig. 5). For intra-sample reproducibility assessment, three keloid (numbered 1 to 3) and three normal samples (numbered 4 to 6) were randomly selected. Each sample was analyzed using the RM method at five randomly chosen nonoverlapping regions in a double-blind manner. Resultant RM mean and RM standard deviation (SD) values are shown in Figs. 5(a) and 5(b), with the coefficient of variation (CV) of each sample annotated on each bar. All CV values were below 9%, indicating excellent reproducibility across both tissue types (CV<15% as a reference of good reproducibility).^33^ To assess inter-operator variability, another set of three keloid (numbered 7 to 9) and three normal samples (numbered 10 to 12) was randomly chosen. Each sample was quantified by three independent operators (A, B, and C) under double-blind conditions. The RM mean and RM SD values measured by three operators are presented in Figs. 5(c) and 5(d). The intra-class correlation coefficient (ICC) was 0.998 for RM mean and 0.997 for RM SD (both 95% confidence interval). Besides, Tukey’s post-hoc tests revealed no statistically significant differences among operators for either tissue type (all p>0.05). Both the ICC results and Tukey’s test supported high consistency in RM quantification across operators. These findings demonstrated high intra-sample reproducibility and low intra-observer variability of biomarkers RM mean and RM SD as results of RM quantification, reinforcing our method’s reliability for further utility.

### 3.3 Importance Ranking and Feature Subset Optimization by XGBoost

**Fig. 6:**
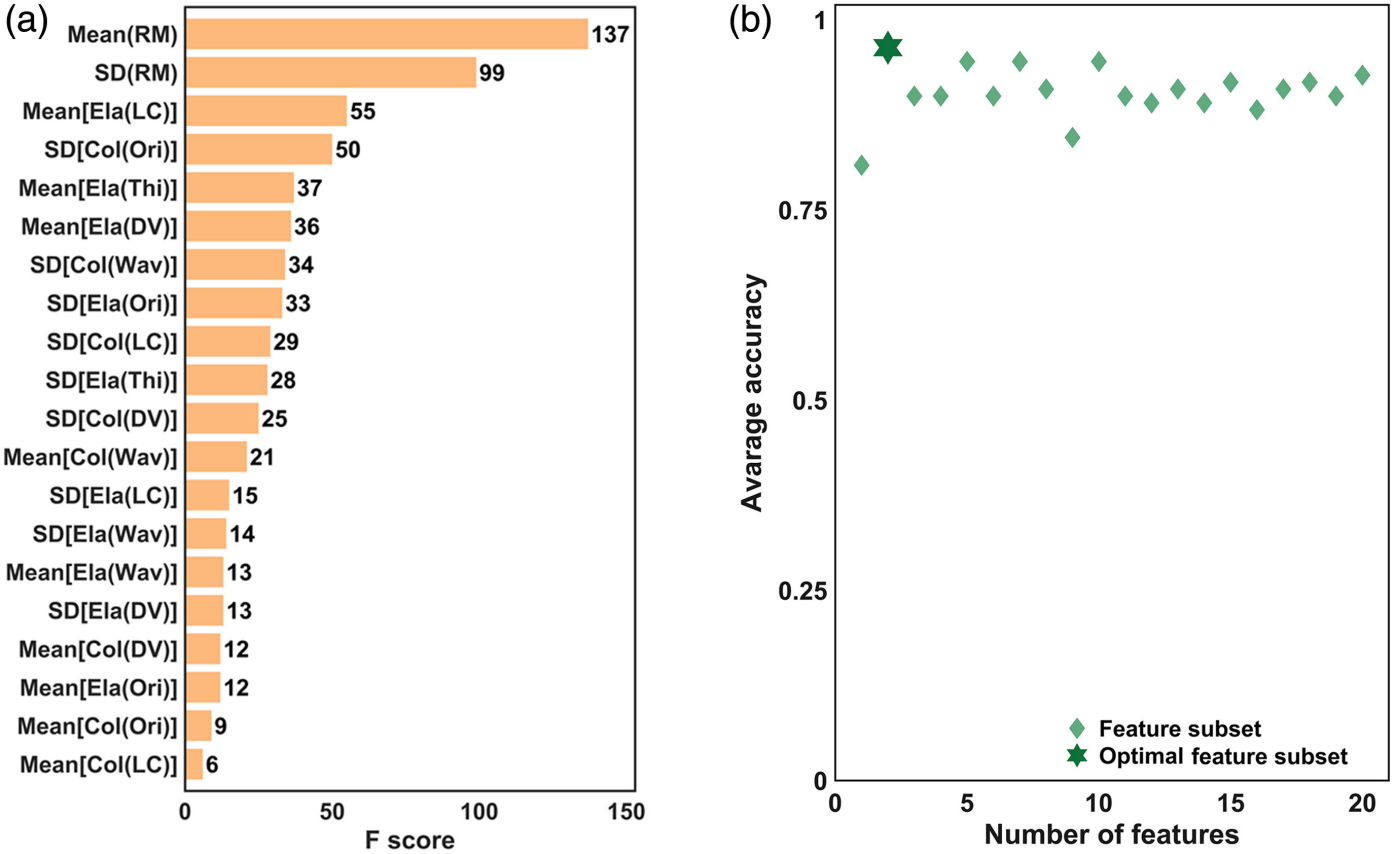
Assessments on 20 features through the XGBoost method. (a) Rank of feature importance by F score of XGBoost output. (b) Scatter plot of average accuracy calculated based on each top-n-ranked feature subset, with the accuracy of the optimal feature subset highlighted as the hexagon token at 96.4% when the top two features were included. SD, standard deviation; Ela, elastin; Col, collagen; RM, resemblance metric; LC, local coverage; Ori, orientation; Thi, thickness; DV, directional variance; Wav, waviness.

After quantitative MPM imaging, we calculated the mean and standard deviation (SD) of all the feature maps, including local coverage, orientation, directional variance, and waviness of both fibers, thickness of elastin fibers, and the resultant RM. A total of 20 statistical indicators were acquired for each one in 108 samples (47 normal, 27 adjacent, and 34 keloid scar). Based on the extracted statistics, we conducted the XGBoost method to assess the importance of each feature and sorted the importance of these features [Fig. 6(a)]. Hyperparameters (Table S2 in the Supplementary Material) were optimized via Bayesian methods to achieve optimal feature subset decision for subsequent tissue classification modeling. As a result, RM mean and RM SD ranked the top two important features. Next, we implemented an incremental feature selection based on the importance ranking result to identify the optimal feature subset. The process evaluated 20 feature subsets (each subset containing the top n ranked features) in order, with each subset evaluated with five repeated runs of XGBoost classification using stratified fivefold cross-validation. Initial classification using only the top-ranked feature (RM mean) achieved 80.9% average accuracy, whereas incorporating the top two features yielded significantly improved classification accuracy (96.4%) as the optimal feature subset [Fig. 6(b)].

### 3.4 RM Comparison among Normal, Adjacent, and Keloid Scar Tissues

**Fig. 7:**
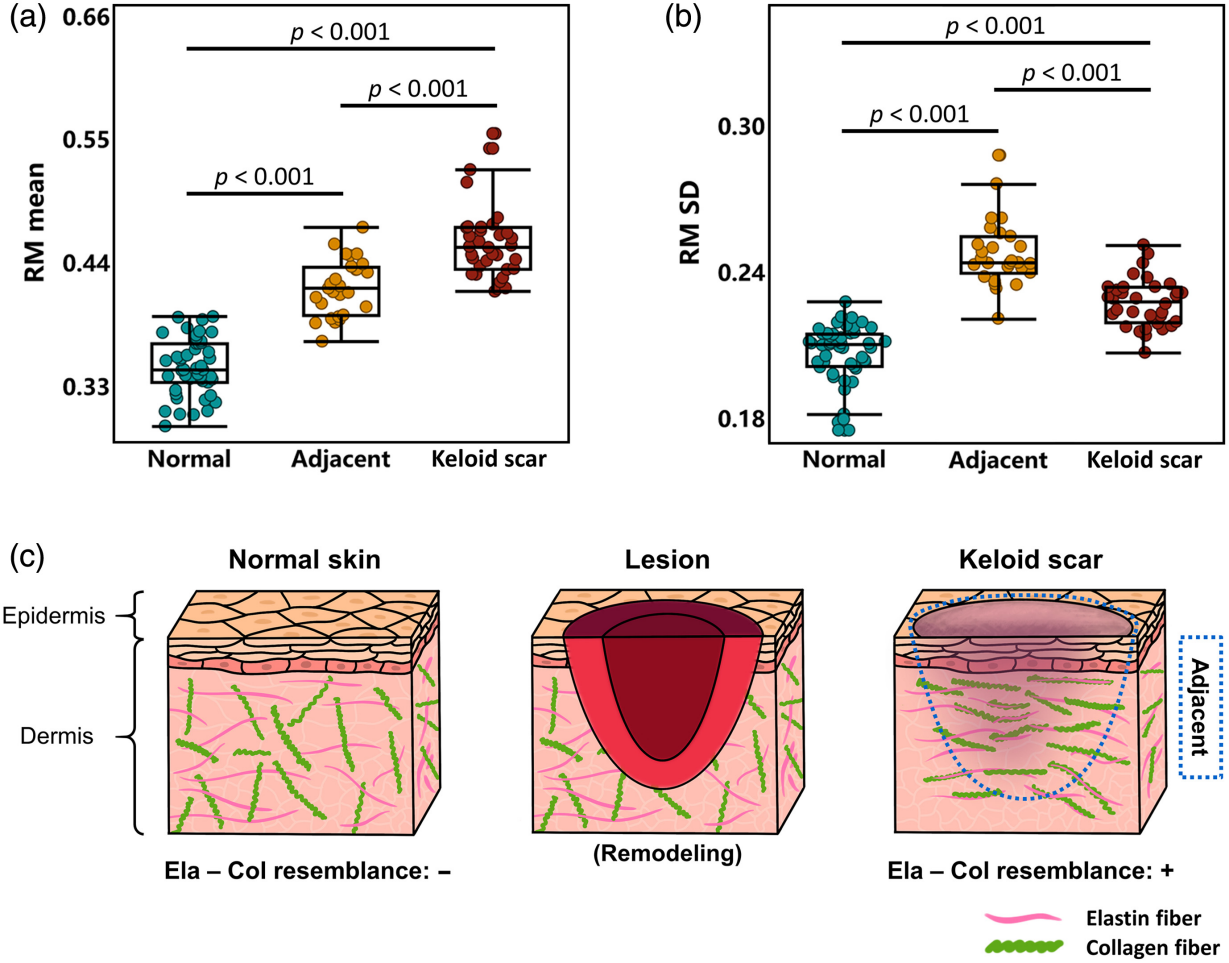
Performance of RM in distinguishing normal, adjacent, and keloid scar tissues. (a) Boxplot of RM mean. In total, 47 normal, 27 adjacent, and 34 keloid scar samples were included in this study. (b) Boxplot of RM SD. (c) Schematic illustrating that the increasing RM reflects elastin and collagen resembling within the ECM of keloid scar, as a result of disordered cutaneous wound healing. RM, resemblance metric; SD, standard deviation; Ela, elastin; Col, collagen.

The boxplots of the optimal feature subset, RM mean and RM SD, are shown in Fig. 7, with statistical significance annotations denoted on the top. For RM mean, Fig. 7(a) demonstrates that keloid scar tissues had an overall high level, whereas normal tissues showed the lowest level of elastin–collagen resemblance, indicating that the abnormal remodeling of elastin and collagen fibers induced by scar lesion resulted in a collective distribution of high RM level. For RM SD, Fig. 7(b) indicates that adjacent tissues exhibited the highest variation level, indicating that adjacent tissues had a highly heterogeneous distribution in elastin–collagen fibrous resemblance. For both RM mean and RM SD in the optimal feature subset, all inter-group comparisons showed p<0.001 (ANOVA with Tukey post-hoc test) in significance.

Based on the elastin–collagen RM analysis among normal, adjacent, and keloid scar tissues reflected by RM mean and RM SD, we drew a schematic to interpret the elastin–collagen resemblance with regard to ECM remodeling [Fig. 7(c)]. To sum up, elastin and collagen fibers within normal tissues exhibited rather independent distributions that the overall inter-heterotypic-fibrous resemblance concerning morphology and organization was low. However, following the formation of skin lesions and subsequent (aberrant) ECM remodeling, the elastin–collagen resemblance level elevated in keloid scars, and the resemblance displayed a more heterogeneous distribution in the adjacent areas. Statistical analysis results and relevant analysis on the variation of elastin–collagen resemblance supported the optimal feature subset’s classification potential to be applied with other disease models (not limited to skin disease models) of RM feature.

### 3.5 Classification Performance of RM-Based K-Nearest Neighbor Model

**Fig. 8:**
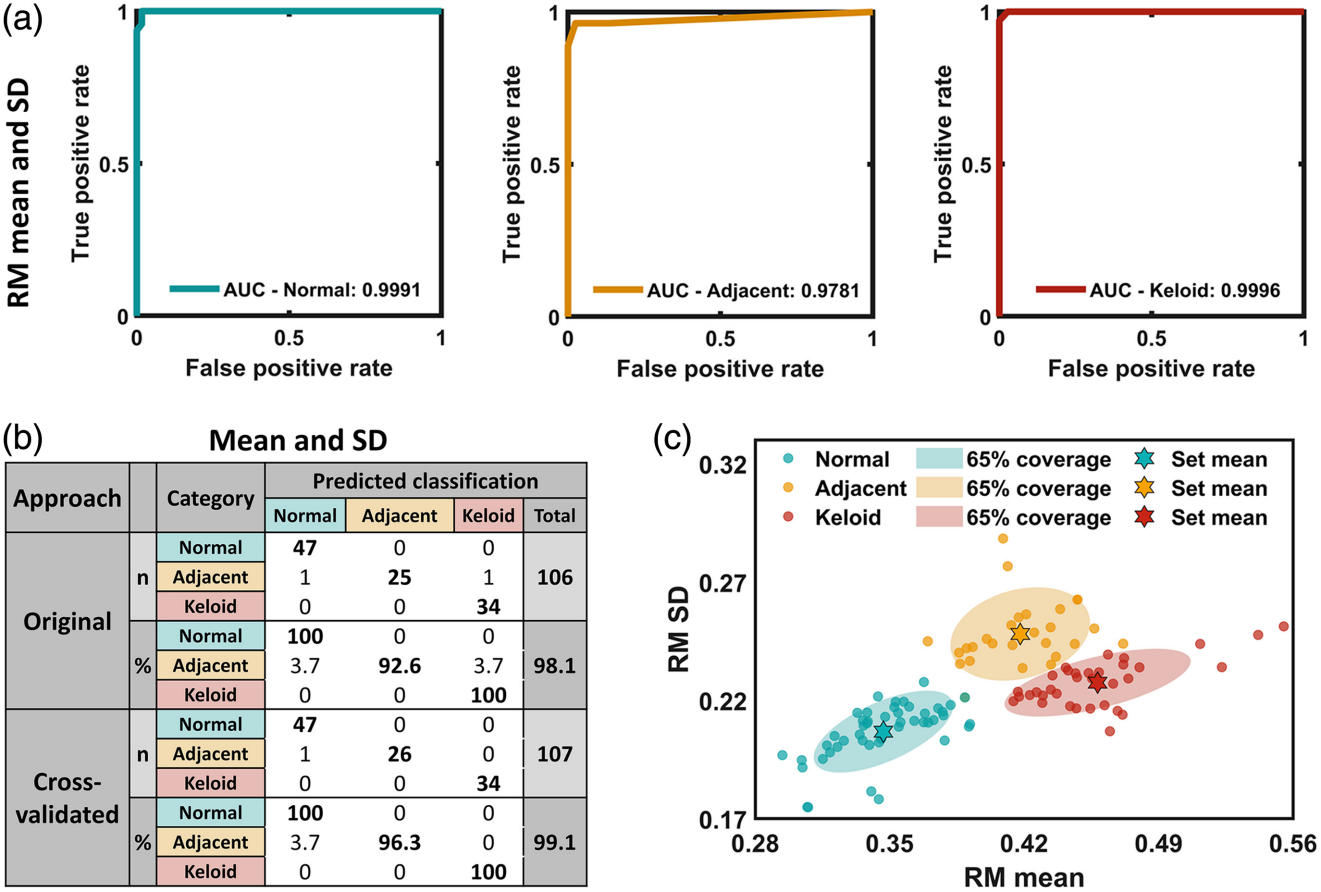
Classification of normal, adjacent, and keloid scar tissues using the K-nearest neighbor model with a combination of RM mean and RM SD via the leave-one-out technique. (a) Receiver operating characteristic (ROC) curves with area under the curve (AUC) values labeled in the graph. (b) Original and cross-validated accuracy values with details of classification results. (c) Scatter plot on normal-adjacent-keloid classification using a combination of RM mean and RM SD features with 65% coverage ellipsoid for each category. RM, resemblance metric; SD, standard deviation.

The final classification model was implemented using a K-nearest neighbor (KNN) machine learning model using a one-vs.-rest technique for normal, adjacent, and keloid scar classification. The key hyperparameter k (number of neighbors) was optimized to be six via comprehensive leave-one-out (LOO) cross-validation on the 108 samples. Diagnostic performance was validated by receiver operating characteristic (ROC) curves shown in Fig. 8(a), revealing exceptional classification capability with area under the curve (AUC) values of 0.9991 for normal tissue identification, 0.9781 for adjacent tissue identification, and 0.9996 for keloid scar identification. Meanwhile, the original classification accuracy (OCA) was 98.1%, and the cross-validated classification accuracy (CVCA) with the LOO technique was 99.1% [Fig. 8(b)]. The 2D scatter plot of RM mean and RM SD is shown in Fig. 8(c), with 65% coverage ellipsoid and the set mean of each category illustrated.

**Fig. 9:**
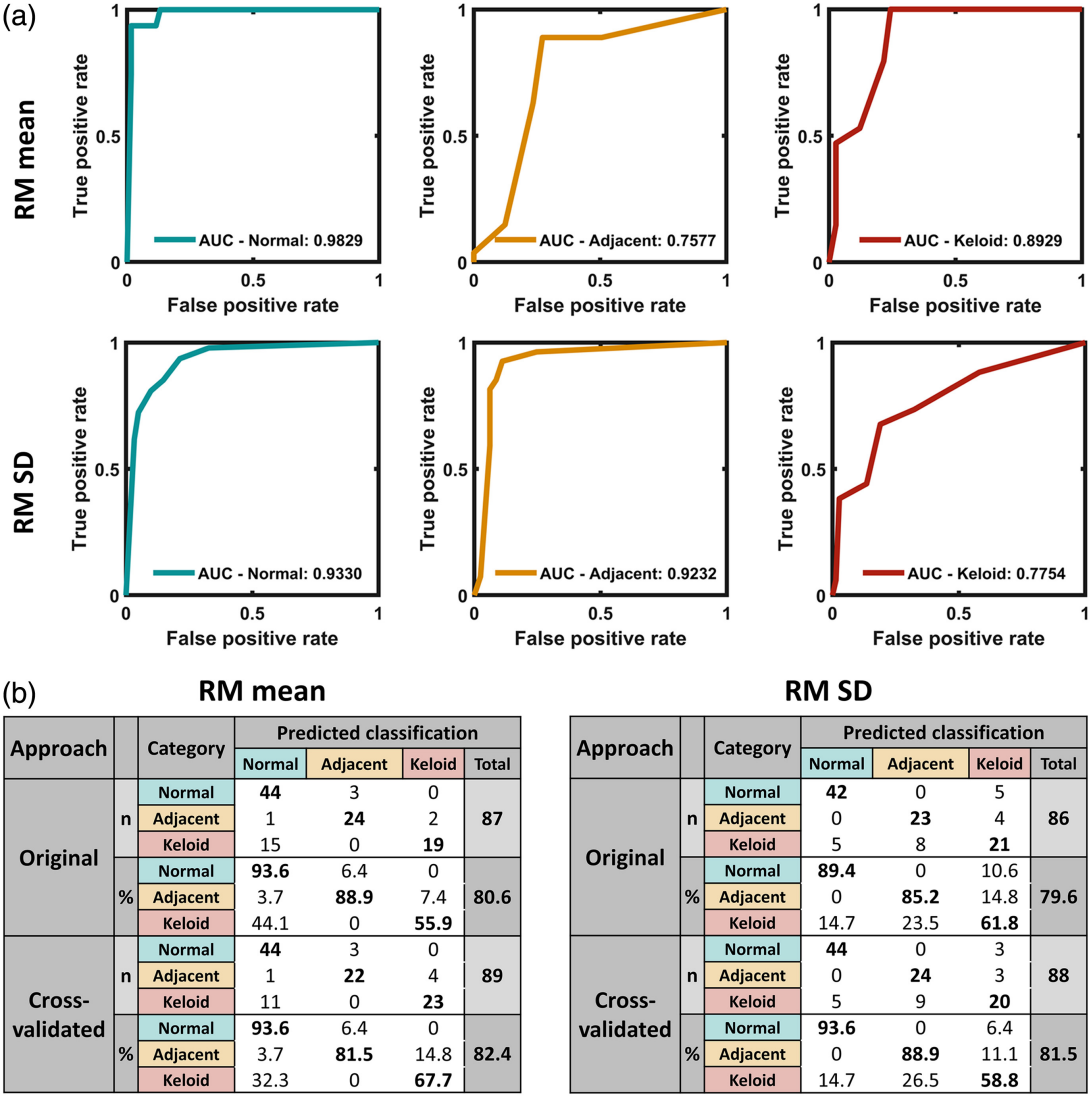
Classification of normal, adjacent, and keloid scar tissues using the K-nearest neighbor model with either RM mean or RM SD via the leave-one-out technique. (a) Receiver operating characteristic (ROC) curves with area under the curve (AUC) values labeled in the graph for each category with either RM mean (top) or RM SD (bottom). (b) Original and cross-validated accuracy values with details of classification results for either RM mean (left) or RM SD (right). RM, resemblance metric; SD, standard deviation.

It is worth mentioning that the optimal feature combination of RM mean and RM SD as input to the KNN model offered better performance than using either RM mean or RM SD alone, as observed from the receiver operating characteristic (ROC) curves with area under the curve (AUC) values [Fig. 9(a)], as well as the calculated OCA and CVCA results [Fig. 9(b)]. Notably, the entire calculation process was completed within seconds. These results demonstrated a combination of RM mean and RM SD as an optimal feature subset with the KNN model provided robust discriminative power across all three tissue categories.

## 4 Discussion

Keloid scars exhibit persistent growth beyond the original wound boundaries after healing and are accompanied by a series of complications. Current clinical diagnosis relies on destructive, time-consuming, and labor-intensive pathological examination, being subject to observer bias. Due to the invasive nature into surrounding skin, accurately characterizing and differentiating among normal, scar, and the adjacent regions are crucial. At the microscale, signatures reflecting ECM components’ remodeling during aberrant healing from skin lesions can serve as biomarkers for a more insightful understanding of the skin recovery mechanism, thus facilitating tissue distinction. Hence, we developed an automated, rapid, and label-free MPM imaging platform with multiple morphological fiber features to investigate ECM remodeling within the human keloid scar model. In particular, we integrated the morphological parameters of fibrous structures into a new parameter termed RM to reveal the elastin–collagen similarity in morphology and organization. This concept was prompted by a former work targeting investigating the resemblance between distinct cellular cytoskeleton components within cells.^34^ We improved the algorithm by integrating features from additional morphological perspectives, including local coverage, directional variance, and waviness, achieving a more accurate and comprehensive map of elastin–collagen resemblance.

The elastin–collagen relationship is also closely correlated with the function of bio-microstructures. In the porcine arterial wall, the observation that collagen fibers reoriented under mechanical loading while elastin fibers maintained their distribution suggested that elastin was prestretched, thereby imparting intrinsic compressive stress on the collagen network.^35^ A similar conclusion was drawn from human aortas, with both a mechanically interlinked network of elastin and collagen fibers and transversely oriented elastin fibers anchoring the collagen fibers observed.^36^ Another dynamical–mechanical test performed on bovine dermal suggested that spatial assembly of the elastin–collagen fibrous network with physically coupling mechanisms might impact the mechanical response of tissues.^37^ As for human skin tissues, the resilient recoil and anisotropic strain-stiffening properties of skin emerged from the intricate entanglement and mechanical connectivity between the dermis’s fibrous collagen architecture and the elastin network.^38^ These findings hinted at an underlying relationship between the organization of elastin and collagen fibers, especially during the process of recovering from a skin lesion. In this sense, the dysregulated remodeling of these two ECM fibrous components might end up with the altered morpho-structural resemblance level as a result of abnormal scar recovery, and further experiments are needed to validate this possibility in the future.

Compared with existing computer-aided scar analysis approaches that often inadequately address feature extraction, particularly regarding ECM components, we focus on both elastin and collagen fibers that play critical roles in keloid progression as ECM key components, especially the often-overlooked elastin fibers. Quantification of the resemblance level between them constitutes the largely unknown elastin–collagen resemblance in dermatology. In short, taking multiple morphological perspectives into consideration, the resemblance level between them is higher in keloid scar tissues compared with normal and adjacent samples. The quantification results of our newly developed biomarker, RM, provide quantitative support for this finding. Furthermore, the combination of RM mean and RM SD is further identified to be the optimal feature subset over others to identify normal, keloid scar, and adjacent tissues with machine-learning algorithms at high sensitivity. Besides, there were no significant differences observed among the samples’ RM values concerning patients from different age and gender groups. Samples from different anatomical locations also showed similar RM contrast among normal, keloid scar, and adjacent tissues. Consequently, RM establishes the resemblance level of ECM components as a novel perspective for understanding ECM remodeling in pathological conditions.

Another interesting fact of keloid scar is the reported alteration of stiffening along progression. Specifically, compared with a fourfold increase in stiffness of normal scar,^39^ stiffness of keloid tissues is about 10-fold greater than that of normal skin tissues,^23^ with observations from atomic force microscopy (AFM) measurement. Meanwhile, tissues with heightened stiffness, also featured with thicker collagen bundles, are designated as “stiff.”^40^ It is worth noting that collagen fibers account for ∼70% of the weight of dry human dermis,^41^ providing critical structural support as the scaffold of the skin.^42^ However, elastin fibers cover ∼4% of the weight of dry human dermis, providing recoil when applied with deforming stresses.^43^ In other words, collagen offers tissues strength and support, whereas elastin provides elasticity and flexibility.^44^ RM quantification results revealed that elastin–collagen resemblance in keloid scar exhibited a higher level over adjacent and normal tissues, which might also be the attribution of keloid stiffening, in addition to the unbalanced elastin–collagen content ratio resulted by overproduction of collagen.^25^ Meanwhile, the increased heterogeneity of elastin–collagen resemblance indicated by RM SD is also consistent with the adjacent region being the translational zone between the keloid scar and normal skin. In short, our newly developed feature RM provides new interpretation and possibilities for the pathological resolution of keloid scars, potentially helping to enrich the knowledge of this skin abnormality and distinguishing the target treatment areas.

Several limitations should be noted in this study. First, validation across a larger and more diverse patient population, especially concerning ethnicity, will help to improve the pathological understanding of keloid scar formation. Second, though 2D analysis is of advantage in quantification speed, 3D quantitative imaging should be introduced to picture a more anatomically realistic map of feature analysis and improve the sensitivity of our developed machine learning-based classification method for the human keloid scar model. This optimization frame was inspired by a previous work that also used a machine learning-aided incremental feature selection trick to find the optimal feature subset for the classification of scar tissues.^45^ However, with Bayesian optimization and RM establishment, our method yields better results with higher sensitivity. In addition, our study is based on instant MPM *ex vivo* imaging for freshly excised tissues, whereas *in vivo* MPM imaging is of more potential to be translated to clinical scenarios as a truly noninvasive probing approach. In the future, point-to-point analysis between stiffness maps and RM maps is expected to assess our hypothesized speculative correlation between mechanical properties and RM within the human keloid scar model. Furthermore, applying our approach to data across key scar recovery stages, such as the proliferation phase^46^ during wound healing, and comparing the result of which with those from mature keloid scar, would further provide more insights into the understanding of the ECM dynamic remodeling during pathological progression in keloid scar development.

## 5 Conclusion

This study established a quantitative MPM imaging platform for ECM component analysis for the human keloid scar model by leveraging endogenous TPEF/SHG signals of elastin/collagen for the extraction of discriminative morphological features. In particular, we developed a new parameter termed RM to quantify the elastin–collagen resemblance level. To our knowledge, this is the first time that the relationship between these fibers has been quantified at the microscale, with a significantly higher elastin–collagen resemblance level identified in keloid tissues. Using XGBoost and KNN machine-learning algorithms, we validated that an optimal feature subset comprising two derivatives of RM (mean and standard deviation) successfully distinguished among normal, keloid, and adjacent tissues, with superior sensitivity over other morphological feature subsets. In summary, RM provided new insights into ECM fiber alteration during human keloid scar formation, potentially calling for subsequent studies to investigate the correlation between RM variation and altered stiffness during keloid scar formation. We believe RM would serve as a promising optical biomarker in understanding ECM fiber components’ remodeling in a broader range of physiological and pathological conditions.

## Supplementary Material

10.1117/1.JBO.31.3.036005.s01

## Data Availability

Data and code developed in this study are available upon reasonable request to the corresponding authors.
