# Supplementary material for: Decoding elastin–collagen resemblance in keloid scar through label-free imaging and machine learning
Source: J Biomed Opt. 2026 Mar 14;31(3):036005. doi: 10.1117/1.JBO.31.3.036005 (PMC12988766; doi:10.1117/1.JBO.31.3.036005)
Supplement: Supplementary file 1 [file JBO_031_036005_SD001.pdf]

Decoding elastin-collagen resemblance in keloid scar through label-free imaging and machine learning

Chuncheng Wang, Jia Meng, Lingxi Zhou, Lingmei Chen, Shuhao Qian, Rushan Jiang, Changyong Chen, Lu Yang, Lu Chen, Zhihua Ding, Shuangmu Zhuo,* Zhiyi Liu*

*****Correspondence: Zhiyi Liu, liuzhiyi07@zju.edu.cn; Shuangmu Zhuo, shuangmuzhuo@gmail.com

Supplementary Material includes:

Supporting Material Text

Figures S1 to S2

Tables S1 to S2

Supporting Material Text

Details on Section 2.4 Feature extraction

1. Local coverage algorithm

The two-dimensional (2D) local coverage feature measured regional fiber distribution[26]. After intensity normalization, fiber pixels were segmented from background using automatic OTSU thresholding[27], followed by noise removal via connected domain analysis. The 2D local coverage was calculated as:

$$L_{2D} = \frac{\sum_{x=1}^{n}\sum_{y=1}^{n} F(x,y)}{n^2}, \tag{1}$$

where F represents the binary mask of fibrous structure within the square region centered on the target pixel, and n denotes the size of the square region.

2. Orientation algorithm

The 2D fiber orientation was characterized by the azimuth angel θ in 2D flat, spanning 0°-180° (Fig. S1 in the Supplementary Material). Specifically, it was computed by firstly generating an $n \times n$ pixel square centered on the target fiber pixel and then calculating all center-passing vectors weighted by two factors W_1 and W_2:

$$W_1 = \frac{1}{L}, \tag{2}$$

$$W_2 = \sqrt{\frac{1}{3}} - \sqrt{\frac{1}{2}\sum_{i=1}^{3}(a_i - \bar{a})^2}, \tag{3}$$

where W_1 weighted the vector by inverse the vector length L, while W_2 weighted the vector by intensity variation, with a_1, a_2 and a_3 representing intensities of the central pixel and its two symmetric endpoints of each vector, and $\bar{a}$ denoting their average intensity.[28] The

determination of square size n was referred to the estimated fiber diameter. Finally, the azimuthal orientation θ was determined by summing all weighted vectors.

3. Directional variance algorithm

The 2D directional variance feature measured alignment of local fibers (0-1 scale), where 0 denoted perfect parallelism and 1 represented fully random orientation distribution.[29] It was calculated as:

$$V_{2D} = 1 - \sqrt{\bar{C}_{2D}^2 + \bar{S}_{2D}^2}, \tag{4}$$

where:

$$\bar{C}_{2D} = (1/u)\sum_{i=1}^{u} \cos(2\theta_j), \tag{5}$$

$$\bar{S}_{2D} = (1/u)\sum_{i=1}^{u} \sin(2\theta_j), \tag{6}$$

where θ was the orientation described above, and u was the total number of pixels in the neighborhood centering at the chosen pixel.

4. Waviness algorithm

The 2D waviness metric (0-1 range) evaluated fiber curvature, with values closer to 0 representing straighter fibers and higher values indicating greater curvature.[30] For each target pixel, a square region of interest was analyzed by computing orientation differences (δ) between the central pixel and its neighbors, which were converted to absolute values and adjusted to complementary angles when exceeding 90°. The waviness was then determined using the following equation:

$$W_{2D} = \frac{1}{90}\frac{1}{u}\sum_{i=1}^{u} \delta_{\theta_i}, \tag{7}$$

where u was the total number of non-central fiber pixels within the square region.

5. Thickness algorithm

The thickness algorithm estimated pixel-wise fiber diameter in images,[31] and it was applied to elastin fibers exclusively, as collagen fiber entanglement hindered the distinction of single fiber. Based on the fibrous segmentation mask, the method calculated the minimum distance between each fiber pixel and its nearest background pixel, propagated these values adaptively to unify perpendicular estimates, and used smoothing to generate the final thickness map.

Figures

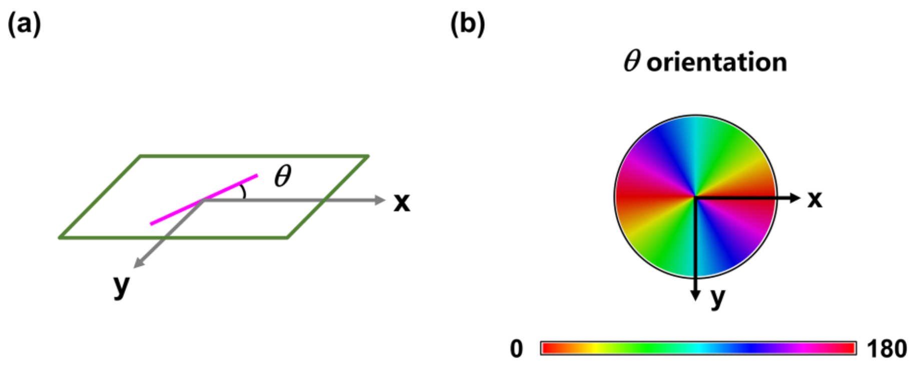

Fig S1. 2D illustration of angles depicting certain orientation of a fiber. (a) Definitaion of azimuthal angle θ used to depict an orientation in a 2D plane. (b) θ map in the xy plane for indication of fiber orientation, together with corresponding color bar.

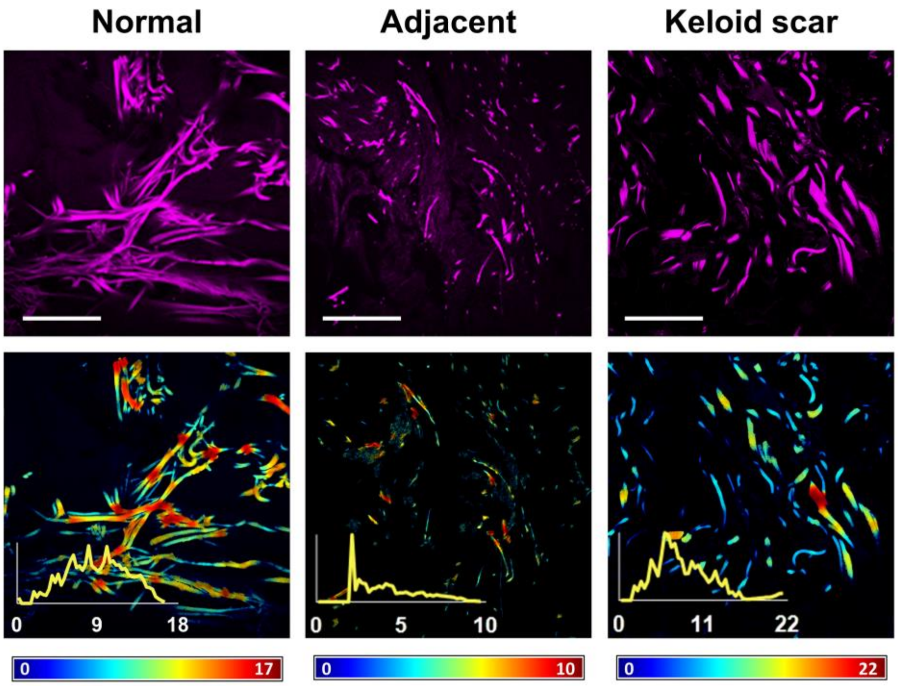

Fig S2. Pixel-wise elastin thickness maps of normal, adjacent and keloid scar samples, with thickness histogram distributions depicted. Scale bar, 50 µm.

Tables

Table S1. Demographic information of samples.

Characteristics		Normal (*n*=47)		Adjacent (*n*=27)		Keloid scar (*n*=34)	
		No.	%	No.	%	No.	%
Age (years)							
	10-20	6	12.8%	5	18.6%	6	17.6%
	21-30	16	34.0%	11	40.7%	12	35.4%
	31-30	13	27.7%	6	22.2%	7	20.6%
	41-50	9	19.1%	3	11.1%	6	17.6%
	51-60	3	6.4%	2	7.4%	3	8.8%
Gender							
	Male	21	44.7%	12	44.4%	14	41.2%
	Female	26	55.3%	15	55.6%	20	58.8%
Sites							
	Face or neck	8	17.0%	4	14.8%	6	17.6%
	Chest	17	36.2%	9	33.4%	11	32.4%
	Shoulder	7	14.9%	5	18.5%	6	17.6%
	Abdomen or back	5	10.6%	3	11.1%	4	11.8%
	Limbs	10	21.3%	6	22.2%	7	20.6%

Table S2. XGBoost hyperparameter values.

Hyperparameter name	Value
max_depth	6
learning_rate	0.13
min_child_weight	2
colsample_bytree	0.8
subsamole	0.6
gamma	0.2
reg_alpha	0
reg_lambda	1
